# A Single-Center Retrospective Analysis of the Diagnostic Efficacy of Transthoracic Echocardiography in the Classification of Patent Ductus Arteriosus

**DOI:** 10.31083/RCM51081

**Published:** 2026-09-04

**Authors:** Minxin Zhang, Chen Qian, Shuang Wang, Tianxing Fang, Bin Wang, Jinping Liu

**Affiliations:** ^1^Department of Cardiovascular Surgery, Zhongnan Hospital of Wuhan University, 430071 Wuhan, Hubei, China; ^2^Hubei Provincial Engineering Research Center of Minimally Invasive Cardiovascular Surgery, 430071 Wuhan, Hubei, China; ^3^Wuhan Clinical Research Center for Minimally Invasive Treatment of Structural Heart Disease, 430071 Wuhan, Hubei, China; ^4^Department of Cardiovascular Ultrasound, Zhongnan Hospital of Wuhan University, Wuhan University, 430071 Wuhan, Hubei, China; ^5^Department of Nuclear Medicine, Zhongnan Hospital of Wuhan University, 430071 Wuhan, Hubei, China

**Keywords:** patent ductus arteriosus, transthoracic echocardiography, digital subtraction angiography, diagnostic efficacy

## Abstract

**Background:**

This study aimed to investigate the diagnostic efficacy of transthoracic echocardiography (TTE) in classifying patent ductus arteriosus (PDA).

**Methods:**

A total of 616 patients diagnosed with PDA underwent TTE. TTE measurements were obtained using the parasternal short-axis view of the great arteries and the suprasternal fossa aortic long-axis view. Using digital subtraction angiography (DSA) as the gold standard, the diagnostic efficacy of TTE for PDA classification was evaluated.

**Results:**

Among true-positive cases correctly diagnosed by both TTE and DSA, the sensitivity for tubular PDA was 100.00%, and the specificity was 50.74%. For funnel-type PDA, the sensitivity was 50.57%, and the specificity was 100.00%. For a window-type PDA, the sensitivity was 60.00%, and the specificity was 100.00%. The internal diameters of the tubular PDA and the pulmonary artery side of the funnel-type PDA measured by TTE showed good agreement with those measured by DSA. In addition, in the suprasternal long-axis view of the ascending aorta (SSLAX-AA) of TTE among true-positive cases, the sensitivity for diagnosing tubular PDA was 100.00%, and the specificity was 75.25%; the sensitivity for funnel-type PDA was 75.51%, and the specificity was 100.00%; the sensitivity for window-type PDA was 66.67%, and the specificity was 100.00%. The diagnostic efficacy of this view was improved compared with the overall TTE, which may be due to the similarity between this view and the DSA view.

**Conclusions:**

Among true-positive cases, TTE has high sensitivity for diagnosing tubular PDA and high specificity for diagnosing funnel-type PDA. Meanwhile, TTE shows good agreement with DSA in measuring key parameters. Furthermore, TTE demonstrates stability in the diagnostic classification of PDA, with no significant influence from operator variability. TTE could serve as a reliable basis for clinical decision-making to aid in the diagnostic classification of PDA.

## 1. Introduction

Patent ductus arteriosus (PDA) is a common congenital heart disease and a pathological condition in which the ductus arteriosus, a fetal vessel connecting the isthmus of the descending aorta to the root of the left pulmonary artery, fails to close normally after birth and remains patent. Epidemiological data indicate that the incidence of PDA in term infants is approximately 2%–4%, accounting for 5%–11% of all congenital heart diseases [1]. The incidence is significantly higher in preterm infants, especially in extremely low-birth-weight infants (<1500 g) or those born before 28 weeks, in whom the incidence of PDA can reach 20%–60% [2,3]. However, the adverse effects of PDA occur across all age groups [4]. Abnormal shunting may lead to a series of hemodynamic disturbances that can gradually increase pulmonary arterial pressure and eventually progress to Eisenmenger syndrome, resulting in the loss of surgical indications. Moreover, the shunt volume depends on the ductal diameter and the pressure difference between the aorta and pulmonary artery [5]. A large left-to-right shunt can increase left ventricular volume load, leading to left atrial and left ventricular enlargement, and even early heart failure. When the diversion of systemic blood flow exceeds the compensatory increase in cardiac output, causing impaired perfusion of important organs such as the intestines, kidneys, and brain, preterm infants may develop a range of complications, including necrotizing enterocolitis, intraventricular hemorrhage, periventricular leukomalacia, cerebral palsy, bronchopulmonary dysplasia, and retinopathy of prematurity [2,6,7,8,9]. Therefore, early and accurate diagnosis of PDA, clear classification, and precise measurement of relevant parameters are crucial for guiding treatment and improving prognosis [10].

According to the morphological characteristics of the ductus arteriosus, the Krichenko classification divides PDA into type A (funnel type), type B (window type), type C (tubular type), type D (complex type), and type E (aneurysmal type) [11]. Preterm and low-birth-weight infants with PDA accompanied by significant hemodynamic abnormalities may receive ibuprofen or indomethacin as first-line agents to promote ductal closure [12,13]. For infants, children, and adults with PDA who do not respond to drug therapy or have contraindications to these medications, interventional or surgical treatment should be considered [14,15,16]. Among these treatments, funnel-type and tubular-type PDAs are often treated with transcatheter closure as the first-line option. Although complex-type and aneurysmal-type ductus arteriosus have irregular morphologies, satisfactory occlusion effects can still be achieved by selecting specialized occluders, such as vascular plugs and Amplatzer II occluders [17,18]. However, a window-type PDA with significant hemodynamic abnormalities is more difficult to treat by interventional closure due to the short, thick ductus and lack of obvious anchoring sites. Specifically, when the ductal diameter exceeds 10 mm or is accompanied by severe hemodynamic abnormalities, the occluder is prone to displacement, and the risk of residual shunt is high. In such cases, surgical treatment is required, and common procedures include thoracoscopic ductus arteriosus clipping and left thoracotomy for ductus arteriosus ligation [19], both of which achieve anatomical closure through direct ligation or clipping of the ductus arteriosus [20]. Therefore, early diagnosis and accurate classification are key factors in selecting appropriate treatment methods and ensuring a favorable prognosis for patients.

Currently, digital subtraction angiography (DSA) is the gold standard for PDA classification. Meanwhile, transthoracic echocardiography (TTE) has become the preferred imaging modality for PDA diagnosis and preoperative evaluation owing to the associated properties, such as non-invasiveness, convenience, and high repeatability. However, the diagnostic efficacy of TTE is affected by factors such as operator experience, patient body type, and acoustic window conditions, and the subsequent accuracy may vary across different clinical scenarios. Therefore, this study aimed to compare the classification results and relevant measurement data obtained by TTE and DSA, systematically evaluate the sensitivity, specificity, and consistency of TTE in PDA classification, and explore the key factors affecting the subsequent diagnostic performance [21]. In addition, we compared the diagnostic efficacy of different TTE views for PDA and discussed possible reasons for the differences in performance among these views.

## 2. Materials and Methods

### 2.1 Study Population

This retrospective study enrolled patients diagnosed with PDA who had surgical indications at Zhongnan Hospital of Wuhan University between January 2015 and January 2025. Based on ductus arteriosus morphology, the patients were divided into tubular-, funnel-, and window-type groups. A total of 616 patients with PDA were included, comprising 298 males (48.38%) and 318 females (51.62%). The patients ranged in age from 2 months to 76 years, with a mean age of 20.12 ± 20.81 years. Among the patients, 237 individuals (38.48%) were infants and young children aged 0–3 years, while 379 cases (61.52%) were older than 3 years. Body mass index (BMI) was normal in 521 patients (84.58%), low in 58 (9.42%), and overweight/obese in 37 (6.00%). Regarding cardiac function, left ventricular ejection fraction (LVEF) was normal in 573 patients (93.02%) and reduced in 43 (6.98%). No patients had other complex congenital heart diseases. Comorbidities involving other systems were present in 96 patients (15.58%), mainly respiratory diseases in 41 (6.66%), digestive diseases in 29 (4.71%), urinary system diseases in 15 (2.44%), and other chronic diseases in 11 (1.79%). No comorbidities were identified in 520 patients (84.42%). Owing to the retrospective nature of the study, informed consent was waived.

Inclusion criteria were as follows: (1) patients diagnosed with PDA and having surgical indications at Zhongnan Hospital of Wuhan University between January 2015 and January 2025; (2) completion of both TTE and DSA examinations before surgery; (3) complete clinical data, including imaging reports and surgical records.

Exclusion criteria were as follows: (1) presence of other complex congenital heart diseases, such as tetralogy of Fallot or transposition of the great arteries; (2) poor TTE or DSA image quality, which affects classification judgment; (3) incomplete clinical data.

The subgroup analyzed using the suprasternal long-axis view of the ascending aorta (SSLAX‑AA) was prespecified in the study protocol. Of the 616 patients in this study, 381 were excluded due to missing sectional images, unclear anatomical structures, or non-diagnostic image quality. All consecutive cases with complete and diagnostically usable SSLAX‑AA images were included, without selective exclusion or enrichment, resulting in 235 cases. All exclusions were based solely on inadequate image quality, and no selective inclusion or exclusion was performed to minimize selection bias (Fig. 1).

**Fig. 1.**
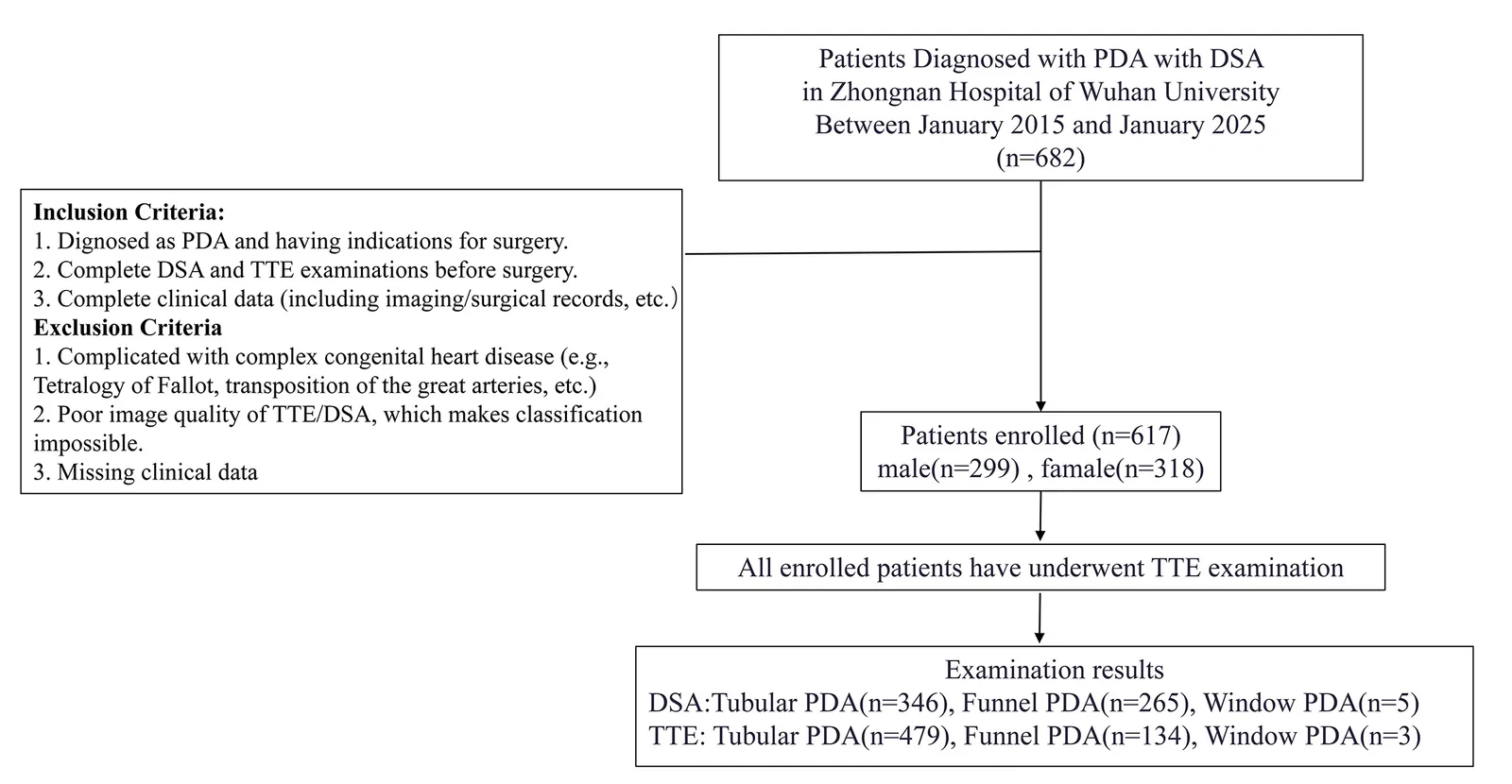
**A flowchart of the patient inclusion and exclusion criteria**. PDA, patent ductus arteriosus; DSA, digital subtraction angiography; TTE, transthoracic echocardiography.

### 2.2 Imaging Diagnostic Methods

All enrolled patients underwent DSA and TTE examinations. A total of 4 qualified full-time echocardiographers participated in the TTE examinations in this study. All four physicians had at least 5 years of clinical experience in the echocardiographic diagnosis of congenital heart disease and had received standardized training in the echocardiographic classification and measurement of PDA. To minimize measurement bias, all investigators were blinded to the DSA results throughout the study. All TTE images were stored independently in the picture archiving and communication system (PACS). Two senior echocardiographers reviewed the results. In cases of disagreement, a chief physician with more than 15 years of professional experience served as the adjudicator, and the adjudication result was used as the final TTE diagnosis for subsequent statistical analysis.

#### 2.2.1 Color Doppler Echocardiography

Color Doppler echocardiography was performed using the Philips EPIQ CVx and EPIQ 7C systems (system firmware version: 6.0.x, Philips Medical Systems Netherlands B.V., Bothell, WA, USA) with dedicated Philips ultrasound imaging software. The hardware manufacturer is Philips Medical Systems Nederland B.V., Veenpluis 6, 5684 PC Best, the Netherlands. The probe frequency was adjusted according to the examination conditions and ranged from 2 to 9 MHz. Patients were placed in the supine or left lateral position to expose the precordial area. Routine scans were performed using the parasternal short-axis view of great arteries (PGASAX) and SSLAX‑AA views. Optimal views with a clear image display were stored. After the ductal structure was clearly visualized, the length, aortic side diameter, and pulmonary artery side diameter were measured in centimeters at the narrowest part during end-systole [22]. For the suprasternal fossa view, the patient was placed supine with the head slightly tilted back. The probe was placed in the suprasternal fossa area, and the sound beam was directed leftward and posteriorly. Based on the long-axis view of the descending aorta, the probe was rotated clockwise toward a sagittal position to detect the ductal structure between the descending aorta and the pulmonary artery, and the subsequent length and diameters at both ends were measured. PDA was classified in both views as follows: tubular type was diagnosed when the diameters at both ends of the ductus were not significantly different; funnel type was diagnosed when the aortic end was wider, and the pulmonary artery end was narrower; window type was diagnosed when the ductus was short and wide, resulting in direct anastomosis between the aorta and the pulmonary artery.

The ultrasonic imaging characteristics of the PGASAX view are as follows: the probe is positioned anterior and inferior to the ductus, the sound beam is nearly parallel to the ductus, and the junction of the ductus and the aorta appears in the short axis of the aorta. The ultrasonic imaging characteristics of the SSLAX‑AA are as follows: the probe is positioned superior and to the right of the ductus, close to the ductus, the sound beam is nearly perpendicular to the ductus, and the junction of the ductus and the aorta appears in the long axis of the aorta [11].

#### 2.2.2 Digital Subtraction Angiography

DSA was performed using the Philips Azurion 7 M20 digital subtraction angiography system (Philips Medical Systems Netherlands B.V., Netherlands), running the Azurion Smart Imaging Control System. Before the examination, the skin condition, history of contrast medium allergy, and other contraindications were evaluated for each patient. After confirming eligibility for the examination, local infiltration anesthesia with 2% lidocaine was administered intradermally, subcutaneously, and around the femoral artery approximately 20–30 minutes before puncture. Routine Seldinger puncture of the anterior wall of the femoral artery was then performed. After successful puncture, a 5–6 F arterial sheath was inserted. Normal saline or heparin sodium was slowly injected through the sheath: normal saline was used when the procedure time was <30 minutes, with 5–10 mL injected every 10 minutes; heparin sodium was used when the operation time was >30 minutes or the patient had a hypercoagulable tendency, at 50–70 U/kg for adults and 70–100 U/kg for children. Subsequently, a pigtail catheter or vertebral artery catheter was inserted and advanced sequentially to the root of the ascending aorta and the main pulmonary artery for angiography. Digital subtraction technology was used throughout the procedure to eliminate interference from bones and soft tissues and to produce dynamic angiographic images. The acquisition rate was set at 25 frames per second, and the complete process of contrast medium filling, flow, and emptying was recorded synchronously. All DSA examinations were performed by radiologists with extensive independent experience in DSA procedures. Images and diagnostic results were stored separately in the system. Finally, all findings were reviewed by a senior physician. Any discrepancies were referred to a chief physician with more than 15 years of clinical experience for arbitration, and the arbitration result served as the final diagnosis of DSA.

### 2.3 Statistical Methods

SPSS 26.0 software was used for statistical analysis. Categorical data in the baseline characteristics are presented as percentages (%). Measurement data with a normal distribution are reported as the mean ± standard deviation ( $\bar{x}$ ± SD), and comparisons between two groups were performed using an independent-samples *t*-test. The effect size is expressed as the mean difference and 95% confidence interval (CI). Measurement data with non-normal distributions are reported as median (interquartile range) [M (P25, P75)], and comparisons between groups were performed using the Mann–Whitney U test. The effect size is expressed as median difference and 95% CI. The count data are summarized as frequencies and percentages, and comparisons between groups were performed using the χ^2^ test or Fisher’s exact test. Qualitative analysis of intraobserver and interobserver diagnostic agreement was performed using Kappa analysis. Kappa values were interpreted as follows: <0.40 indicated poor agreement, 0.40–0.59 indicated moderate agreement, 0.60–0.79 indicated good agreement, and ≥0.80 indicated excellent agreement. A *p*-value ≤ 0.05 was considered statistically significant.

## 3. Results

### 3.1 Diagnostic Efficacy

A total of 616 patients were confirmed to have PDA. DSA classified 346 cases (56.17%) as tubular-type, 265 (43.02%) as funnel-type, and 5 (0.81%) as window-type. TTE classified 479 cases (77.76%) as tubular-type, 134 (21.75%) as funnel-type, and 3 (0.49%) as window-type.

Based on true-positive cases correctly identified by both TTE and DSA, the sensitivity of TTE for diagnosing tubular-type PDA was 100.00% (95% CI: 98.89%–100.00%), and the specificity was 50.74% (95% CI: 44.37%–57.13%). For funnel-type PDA, the sensitivity was 50.57% (95% CI: 44.38%–56.85%), and the specificity was 100.00% (95% CI: 99.18%–100.00%). For the window-type PDA, the sensitivity was 60.00% (95% CI: 22.91%–88.40%), and the specificity was 100.00% (95% CI: 99.51%–100.00%).

In addition, 235 patients who underwent PDA classification using the SSLAX-AA view on TTE were included in the analysis. Compared with DSA, the sensitivity of TTE for diagnosing tubular-type PDA was 100.00% (95% CI: 97.29%–100.00%), and the specificity was 75.25% (95% CI: 66.58%–82.09%). For funnel-type PDA, the sensitivity was 75.51% (95% CI: 66.47%–82.92%), and the specificity was 100.00% (95% CI: 97.33%–100.00%). For the window-type PDA, the sensitivity was 66.67% (95% CI: 18.00%–82.92%), and the specificity was 100.00% (95% CI: 98.41%–100.00%).

In summary, TTE achieved 100.00% sensitivity for diagnosing tubular-type PDA and 100.00% specificity for diagnosing funnel-type and window-type PDA. Therefore, these findings suggest that TTE has high sensitivity and favorable clinical utility for screening tubular‑type PDA (Table 1).

**Table 1.** **Number of cases (%), sensitivity, and specificity of diagnostic classification by total TTE and the TTE in sternal long-axis view of the ascending aorta (SSLAX-AA)**.

Cases		Type A	Type B	Type C
DSA	Cases (%)	265 (43.02%)	5 (0.81%)	346 (56.17%)
Total TTE	Cases (%)	134 (21.75%)	3 (0.49%)	479 (77.76%)
	Sensitivity	50.57%	60.00%	100.00%
	Specificity	100.00%	100.00%	50.74%
TTE in SSLAX-AA	%	31.49%	0.85%	67.66%
	Sensitivity	75.51%	66.67%	100.00%
	Specificity	100.00%	100.00%	75.25%

type A, funnel type; type B, window type; type C, tubular type.

### 3.2 Consistency Analysis of Partial Measurement Parameters between DSA and TTE

The conclusions were derived from a consistency analysis based on true-positive cases that were correctly diagnosed by both TTE and DSA.

(1) In true-positive tubular-type PDA cases, the internal diameter measured by TTE showed high consistency with the data obtained by DSA. The intraclass correlation coefficient (ICC) was 0.96 (95% CI: 0.95–0.97; *p* < 0.001), indicating an extremely strong correlation. Bland–Altman analysis showed a mean bias of –0.01 mm and 95% limits of agreement (LoA) of –0.15 to 0.12 mm (standard deviation of the differences = 0.07 mm), indicating good agreement between the two methods, with differences within the clinically acceptable range (Fig. 2a,b) (Table 2).

**Fig. 2.**
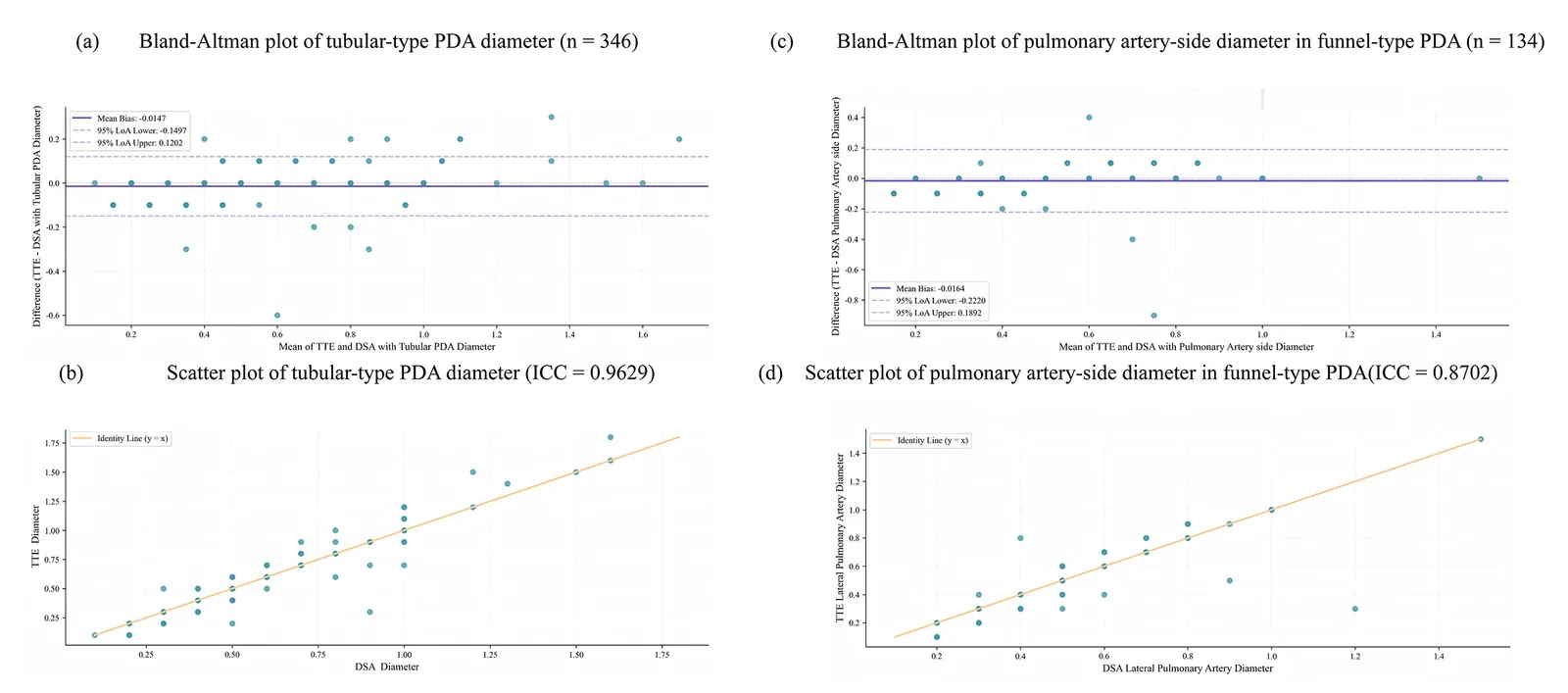
**Consistency analysis of ductal diameter data for true-positive tubular PDA measured by TTE and DSA (a,b), and consistency analysis of pulmonary artery-side ductal diameter data for true-positive funnel-type PDA measured by TTE and DSA (c,d)**.

**Table 2.** **Consistency analysis of diameter measurements between TTE and DSA in true-positive PDA cases diagnosed by TTE**.

Parameters	TTE true-positive tubular PDA diameter	TTE true-positive funnel PDA pulmonary artery diameter
Sample Size	346	134
Bias (mean difference)	–0.01 mm	–0.02 mm
Standard deviation of differences	0.07 mm	0.10 mm
95% LoA lower	–0.15 mm	–0.22 mm
95% LoA upper	0.12 mm	0.19 mm
95% LoA	–0.15–0.12 mm	–0.22–0.19 mm

LoA, limits of agreement.

(2) In true-positive funnel-type PDA cases, the pulmonary artery-side diameter measured by TTE showed good consistency with the data obtained by DSA. The ICC was 0.87 (95% CI: 0.82–0.91; *p* < 0.001), indicating a strong correlation. Bland–Altman analysis showed a mean bias of –0.02 mm and 95% LoA of –0.22 to 0.19 mm (standard deviation of the differences = 0.10 mm), indicating good agreement between the two methods, with differences within the clinically acceptable range (Fig. 2c,d) (Table 2).

(3) In addition, compared with the 346 cases diagnosed as tubular-type by both DSA and TTE, the 131 cases diagnosed as funnel-type by DSA but misdiagnosed as tubular-type by TTE had a significantly longer mean ductal length as measured by DSA; this difference was statistically significant (former group: 0.73 ± 0.37 cm; latter group: mean = 1.16 ± 0.45 cm; *p* < 0.0001) (Table 3).

**Table 3.** **Ductal lengths of tubular and funnel-type PDAs measured by TTE and DSA**.

	Tubular PDA-DSA		Funnel PDA-DSA	
	Length of the ductus arteriosus (cm)	Cases	Length of the ductus arteriosus (cm)	Cases
Tubular PDA-TTE	0.73 ± 0.37	346	1.16 ± 0.45	131
Funnel PDA-TTE	-	0	0.82 ± 0.42	134
Average or sum	0.73 ± 0.37	346	0.97 ± 0.47	265

(4) In addition, 50 patients with tubular PDA and 50 patients with funnel-type PDA were randomly selected. Two independent echocardiographers measured the parameters related to the arterial duct and determined the classification results. We then performed a consistency analysis of the key parameters and a Kappa analysis of the classification results. Furthermore, intraobserver Kappa consistency analysis was performed, in which the same physician, blinded to the previous results, re-evaluated the images after a 5-day interval and provided a new classification. The results showed that the internal diameter data obtained by TTE for tubular-type PDA had good interobserver consistency, with an ICC of 0.83 (95% CI: 0.71–0.42; *p* < 0.001). Bland–Altman analysis showed that the mean difference (A–B) was –0.0020 mm, and the 95% LoA was –0.172 to 0.168 mm (standard deviation of differences = 0.09 mm). The pulmonary artery-side diameter data obtained by TTE in patients with a diagnosed funnel-type PDA also showed good interobserver consistency, with an ICC of 0.94 (95% CI: 0.89–0.96; *p* < 0.001). Bland–Altman analysis showed that the mean difference (A–B) was 0.07 mm, and the 95% LoA was –0.14 to 0.28 mm (standard deviation of differences = 0.11 mm). The aortic-side diameter data obtained when TTE diagnosed funnel-type PDA also had good interobserver consistency, with an ICC of 0.96 (95% CI: 0.93–0.98; *p* < 0.001). Bland–Altman analysis showed that the mean difference (A–B) was 0.01 mm, and the 95% LoA was –0.10 to 0.12 mm (standard deviation of differences = 0.06 mm). The interobserver Kappa coefficient for PDA classification by TTE between the two echocardiographers was 0.92 (95% CI: 0.89–0.95; *p* < 0.001), indicating excellent agreement. The intraobserver Kappa coefficient for a single physician was 0.96 (95% CI: 0.91–0.98; *p* < 0.001), demonstrating excellent diagnostic reproducibility. Both the key parameters and the classification results confirmed that TTE-based PDA classification in this study was highly stable and minimally affected by interoperator variability (Fig. 3).

**Fig. 3.**
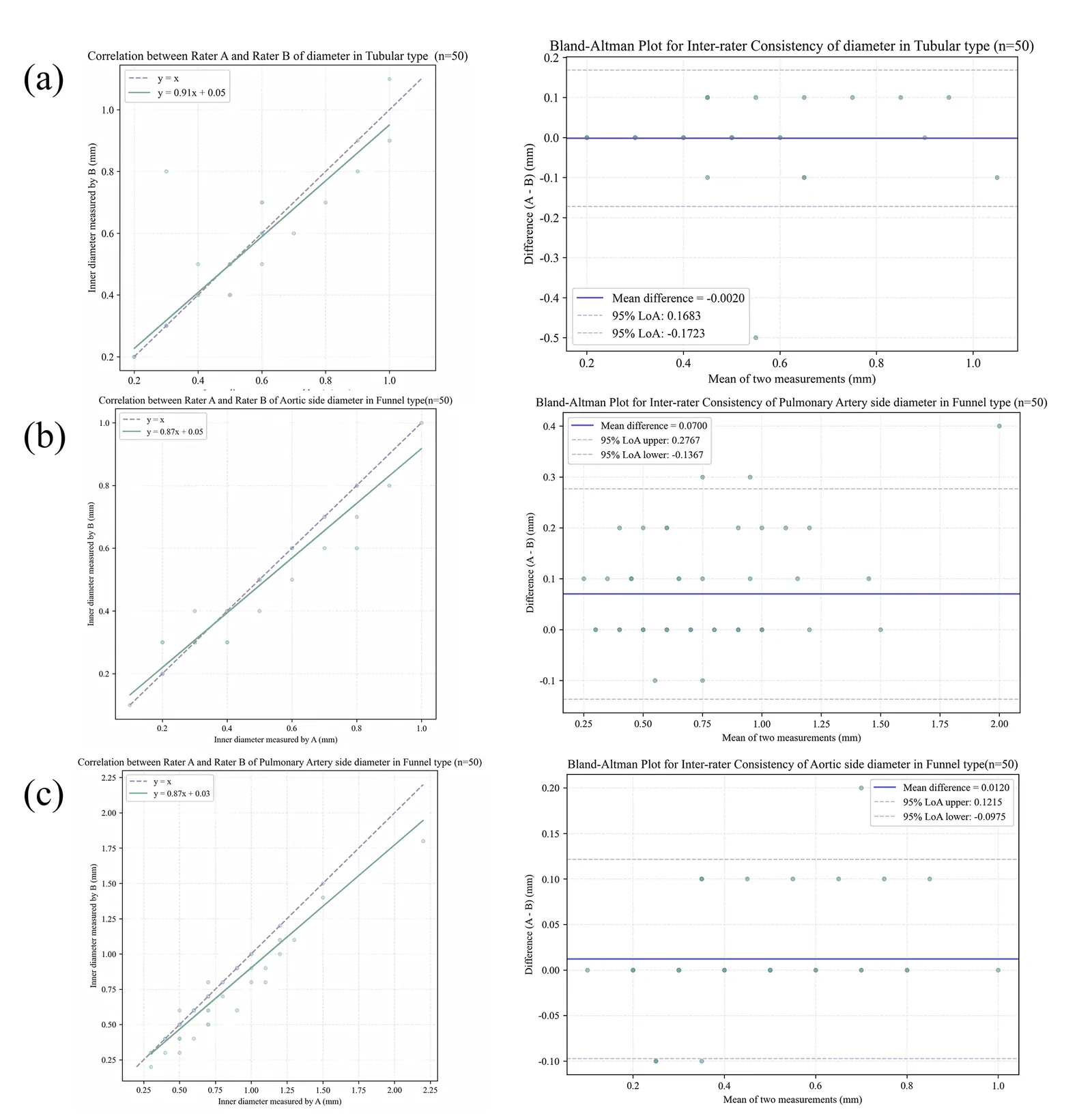
**Interobserver consistency analysis of the tubular PDA diameter (a), the pulmonary artery diameter of funnel-type PDA (b), and the aortic diameter of funnel-type PDA (c), as measured by TTE**.

### 3.3 Age Difference Between Funnel-Type and Non-Funnel-Type PDA Cases Confirmed by the Gold Standard

Among patients with PDA diagnosed by DSA, the mean age of patients with non-funnel-type PDA was significantly lower than that of patients with funnel-type PDA. This difference was statistically significant (independent-samples *t*-test, *p* < 0.0001) (non-funnel-type group: mean = 15.86, SD = 18.17; funnel-type group: mean = 25.78, SD = 22.72).

## 4. Discussion

Despite the above advantages, compared with DSA, TTE still has a certain rate of misdiagnosis and missed diagnosis for tubular-, funnel-, and window-type PDAs. The main possible reason is that the aortic-side diameter measured by TTE is likely smaller than the true value, leading to the misdiagnosis of some funnel-type PDAs as tubular-type PDAs. This difference stems from two major aspects. The first is the geometric relationship among the ultrasound beam, the ductus arteriosus, and the aorta. For the aortic-side diameter, in the SSLAX‑AA view, the ultrasound beam is almost perpendicular to the ductus arteriosus, allowing relatively accurate measurement of the aortic-side ductal diameter (Fig. 4a). In the PGASAX view, the probe is located anterior and inferior to the ductus arteriosus, and the ultrasound beam is almost parallel to the ductus arteriosus. Only when the ultrasound beam passes exactly through the diameter plane at the junction of the ductus arteriosus and the aorta can the maximum aortic-side diameter be obtained. Once the ultrasound beam deviates in any direction, the measured aortic-side diameter of the ductus arteriosus will be smaller than the true value (Fig. 4b), leading the examiner to identify a funnel-type ductus arteriosus as a tubular type. Moreover, we found that the average length of the ductus arteriosus in funnel-type PDA was greater than that in tubular-type PDA; the difference was statistically significant (funnel-type PDA group: mean = 1.16 cm, SD = 0.45 vs. tubular-type group: mean = 0.73 cm, SD = 0.37; *p* < 0.0001), which makes it more difficult for the ultrasound beam to pass accurately through the diameter plane at the junction of the ductus arteriosus and the aorta, further increasing the possibility of error. However, compared with the SSLAX‑AA view, in the PGASAX view, the pulmonary artery is displayed in the long axis, and the pulmonary artery ductal diameter can be fully visualized along this axis, allowing relatively accurate measurement. This view also shows a certain similarity to the gold-standard DSA, consistent with our experimental results showing that the diagnostic efficacy of TTE in the SSLAX-AA view was greater than in the PGASAX view. The second source of measurement error is the influence of the incident angle of the ultrasound beam on the visualization of the target structure. The ultrasound echo signal intensity follows the law of reflection: when the sound beam is incident perpendicularly to the interface, the intensity of the reflected wave is maximal, and the signal is clearest (Fig. 4c); when the incident angle deviates from 90°, the intensity of the reflected wave decreases as the angle increases, and the signal gradually becomes blurred. Therefore, when the ultrasound beam scans perpendicular to the long axis of the ductus arteriosus, the beam can fully transect the lumen, forming a standard cross-sectional image that clearly shows the lumen diameter, morphology, and wall thickness, thereby avoiding morphological distortion and providing an accurate basis for classification. When the ultrasound beam forms an angle with the ductus arteriosus, the beam produces an oblique section and distortion, and may also lead to blurred lumen boundaries due to the partial volume effect [23]. Meanwhile, the intensity of the reflected wave will decrease as the incident angle deviates from 90° (Fig. 4d), further reducing recognition of the lumen boundaries. Simply, morphological distortion and signal attenuation from angled scanning may interfere with the judgment of the examiner of the true morphology of the ductus arteriosus, ultimately leading to misclassification.

**Fig. 4.**
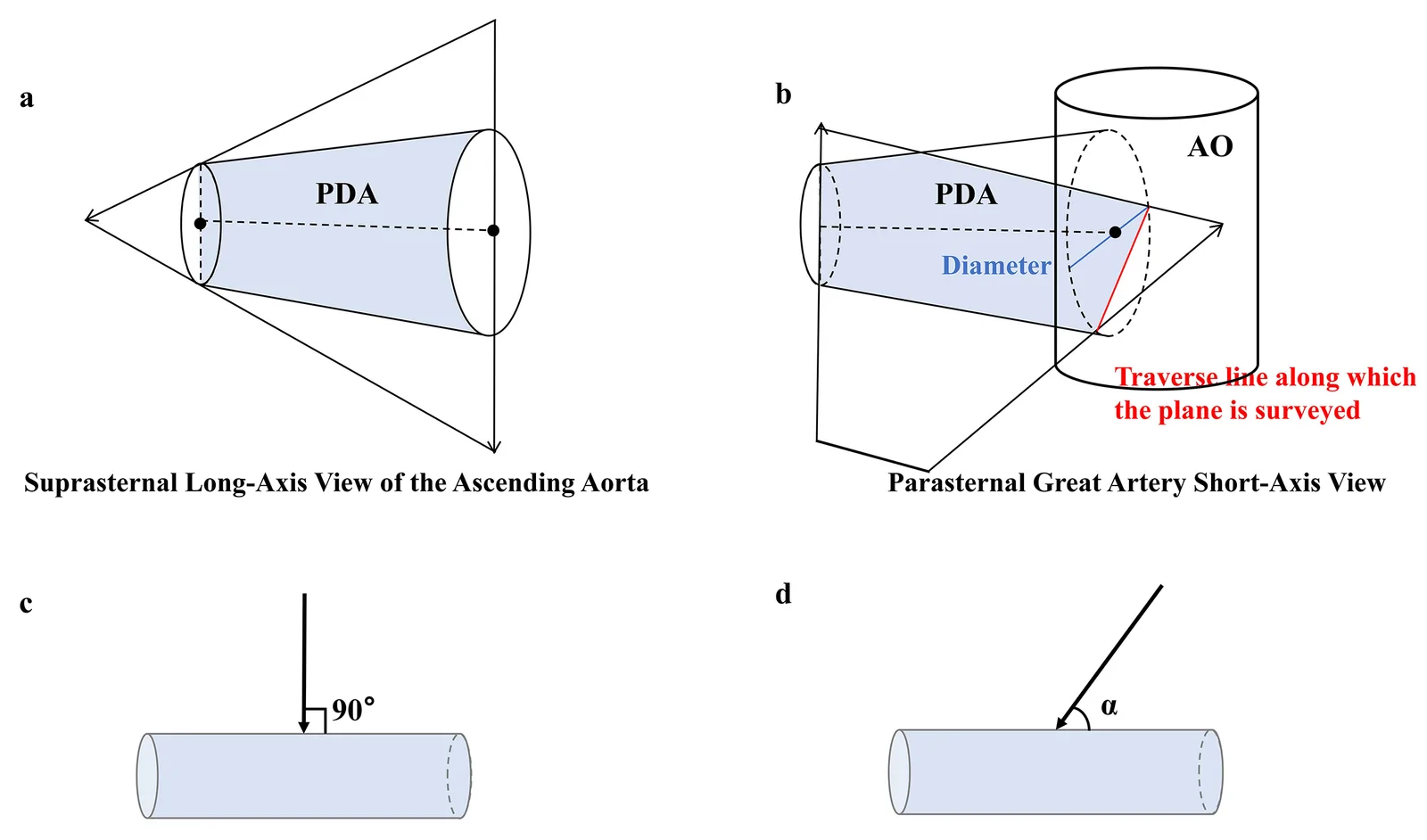
**Schematic diagram of scanning planes. **Schematic diagram illustrating the ultrasound beam scanning the PDA in SSLAX-AA (a). Schematic diagram of PDA scanning via PGASAX. Measurement errors may occur when the ultrasound beam does not pass through the diameter plane; AO: aorta (b). Schematic diagram showing that the ultrasound beam forms a 90° angle with the luminal axis of the PDA under ideal conditions (c). During actual scanning, an included‑angle α exists between the ultrasound beam and the ductal axis, and the angular deviation can trigger measurement errors of the PDA inner diameter (d).

### Limitations

Furthermore, the number of window-type PDA cases included in this study was small. Although excellent specificity was also demonstrated for diagnosing window-type PDA, bias due to the small sample size cannot be excluded. Therefore, the relevant results for window-type PDA were not included in the conclusions of this study. In addition, dumbbell-type and aneurysmal-type PDA were not included. Hence, future studies should include these rare types to further explore the diagnostic efficacy of TTE compared with DSA for such PDAs, and to investigate whether concomitant cardiac diseases affect the diagnosis and classification of PDA by TTE. Moreover, the study population comprised only individuals with surgical indications and complete measurement data from both TTE and DSA. Individuals without obvious hemodynamic abnormalities and those receiving only conservative medical treatment were not included. Additionally, the SSLAX-AA subgroup was based on prespecified criteria and included all patients with interpretable images; however, image availability may have introduced mild selection bias, which should be considered in clinical application. Furthermore, we emphasize that the measurement consistency between TTE and DSA was evaluated only in true-positive cases; therefore, the results should not be generalized to the full diagnostic spectrum. This study was a single-center retrospective study. Although the sample size was relatively large, all cases came from a single medical center, and the clinical characteristics and diagnostic and treatment protocols were specific to that center, which may limit the generalizability of the results to other centers. Therefore, the cohort in this study was selectively defined, which may limit the extrapolation of the results. Accordingly, including a more representative sample of PDA cases and conducting analyses of relevant influencing factors will be the focus of our future work. The overall diagnostic performance of TTE in the entire case cohort still needs to be further validated in larger, more representative samples and in multicenter, prospective studies.

## 5. Conclusions

Accurate diagnosis, classification, and measurement of PDA parameters are of great significance for selecting appropriate treatment strategies. Many previous studies have investigated the use of TTE in diagnosing PDA or guiding PDA intervention [24]. As early as the 1980s, some teams began discussing the sensitivity and specificity of different TTE views for diagnosing PDA [25]. A study by Xiaoyong Zhang et al. [26] in 2012 showed that different TTE views did not differ in PDA detection. Thus, this study aimed to compare the sensitivity and specificity of TTE and DSA in PDA classification. The results showed that TTE demonstrated high sensitivity for tubular PDA and high specificity for funnel-type PDA among true-positive cases correctly diagnosed by both TTE and DSA, with favorable measurement consistency for key anatomical parameters and good inter- and intraobserver agreement. In summary, the high sensitivity of TTE for tubular morphology and high specificity for funnel morphology, together with good inter- and intraobserver agreement for key parameters among true‑positive cases, partly indicate that TTE can provide reliable information for PDA classification. Combined with the advantages of TTE, including non-invasiveness, simplicity, cost-effectiveness, and good reproducibility, the approach may provide valuable references for PDA morphological classification, preoperative parameter measurement, and treatment strategy selection, and could serve as a reliable basis for clinical decision-making to help improve patient prognosis.

## Data Availability

The dataset has been uploaded to Science Data Bank (ScienceDB). The access link is provided as follows: https://www.scidb.cn/s/rQB3Ej.
